# Supplementary material for: Exercise-Based Interventions in Hemodialysis Patients: A Systematic Review with a Meta-Analysis of Randomized Controlled Trials
Source: J Clin Med. 2019 Dec 24;9(1):43. doi: 10.3390/jcm9010043 (PMC7019709; doi:10.3390/jcm9010043)
Supplement: Supplementary file 1 [file jcm-09-00043-s001.pdf]

Supplementary Table S1 *Detailed characteristics of included studies selected for meta-analysis and relevant outcomes*

Study	Population			Training variables	Outcome measures	Results
	Sex;<br>Age (years)<br>[mean ± SD]	Time on dialysis (months)	Sample size	Training program	Outcome measure	
Deligiannis 1999[37]	M and F;<br><br>Ex<br>48 ± 12<br>Con<br>48 ± 11	Ex<br>75.6 ± 36.0<br><br>Con<br>74.4 ± 43.2	Ex (n = 30)<br><br>Con (n = 30)	Ex: non-dialysis days;<br>10-minute warm-up (bicycling and/or walking);<br>a 50-minute aerobic exercise program (calisthenics, steps, swimming, or ball games);<br>a 20-minute stretching and low-weight resistance program;<br>a 10- minute cool-down period (bicycling and/or walking);<br>6 months;<br>3 days/week;<br>90 min;<br>60 – 70% HRmax<br><br>Con: usual care	VO_2max	Ex: 41.0 % ↑*<br>Con: no changes
Afshar 2011[60]	M;<br><br>Ex<br>50.71 ± 21.06<br>Con<br>53 ± 19.4	Ex<br>25.71 ± 7.61<br><br>Con<br>24.86 ± 15.44	Ex (n = 14)<br><br>Con (n = 14)	Ex: cycling during HD;<br>8 weeks;<br>3 days/week;<br>20 min;<br>12 - 15 RPE<br><br>Con: usual care	CRP	Ex: 83.2 % ↓**<br>Con: 1.2 % ↑
Wu 2014[26]	M and F;<br><br>Ex<br>45<br>Con<br>44	Ex<br>55.5 ± 37.3<br><br>Con<br>39.8 ± 29.7	Ex (n = 34)<br><br>Con (n = 35)	Ex: cycling during HD;<br>12 weeks;<br>3 days/week;<br>15 min;<br>12 – 16 RPE<br><br>Con: simple stretching exercises;<br>12 weeks;<br>3 days/week;<br>10–15 min	6MWT<br><br><br>STS-10	Ex: 8.09 % ↓*<br>Con: 4.77 % ↓<br><br>Ex: 5.7 % ↓*<br>Con: 5.0 % ↓
Bohm 2014[27]	M and F;<br><br>Ex<br>52 ± 14.5<br>Con<br>53 ± 16.9	Ex<br>37 ± 69<br><br>Con<br>21 ± 30	Ex (n = 27)<br><br>Con (n = 26)	Ex: cycling during HD;<br>12 weeks;<br>3 days/week;<br>45 min;<br>13 RPE<br><br>Con: walking program at home	$peakVO_2$	Ex:<br>after 12 weeks:<br>9.89 % ↑<br>after 24 weeks:<br>no change<br><br>Con:<br>after 12 weeks:<br>3.31 % ↓

					 <br><br>6MWT	after 24 weeks:<br>1.66 % ↑<br><br><br>Ex:<br>after 12 weeks:<br>6.58 % ↑<br>after 24 weeks:<br>3.96 % ↑<br><br>Con:<br>after 12 weeks:<br>1.67 % ↑<br>after 24 weeks:<br>0.05 % ↓
Groussard 2015[28]	M and F;<br><br>Ex<br>66.5 ± 4.6<br>Con<br>68.4 ± 3.7	Ex<br>36.6 ± 8.2<br><br>Con<br>41.2 ± 8.1	Ex (n = 8)<br><br>Con (n = 10)	Ex: cycling during HD;<br>3 months;<br>3 days/week;<br>30 min;<br>55%–60% peak power<br><br>Con: usual care	6MWT<br><br><br><br><br>peakVO_2	Ex: 23.15 % ↑**<br>Con: 7.98 % ↑<br><br><br><br>Ex: 2.72 % ↓<br>Con: 14.18 % ↑
Hristea 2016[61]	M and F;<br><br>Ex<br>68.5 ± 13.97<br>Con<br>70.8 ± 15.18	Ex<br>139<br><br>Con<br>96	Ex (n = 10)<br><br>Con (n = 11)	Ex: cycling during HD;<br>6 months;<br>3 days/week;<br>30 min;<br>3 RPE (modified Borg scale)<br><br>Con: usual care	6MWT<br><br><br><br>CRP	Ex: 21.8 % ↑**<br>Con: 18.8 % ↓<br><br><br>Ex: 30.6 % ↓**<br>Con: 19.8 % ↓
Liao 2016[29]	M and F;<br><br>Ex<br>62 ± 8<br>Con<br>62 ± 9	Ex<br>71 ± 46<br><br>Con<br>83 ± 71	Ex (n = 20)<br><br>Con (n = 20)	Ex: cycling during HD;<br>3 months;<br>3 days/week;<br>20 min;<br>13-14 RPE<br><br>Con: usual care	CRP<br><br><br>6MWT	Ex: 37.6 % ↓*<br>Con: 0.81 % ↓<br><br>Ex: 11.1 % ↑*<br>Con: 1.1 % ↓
Cheema 2007[30]	M and F;<br><br>Ex<br>60.0 ± 15.3<br>Con<br>65.0 ± 12.9	Ex<br>39.6<br><br>Con<br>19.2	Ex (n = 24)<br><br>Con (n = 25)	Ex: progressive resistance training;<br>two sets of eight repetitions of 10 exercises;<br>during HD;<br>12 weeks;<br>3 days/week;<br>45 min;<br>15 – 17 RPE<br><br>Con: usual care	6MWT<br><br><br><br>CRP	Ex: 3.36 % ↑<br>Con: 0.74 % ↓<br><br><br>Ex: 10.26 % ↓*<br>Con: 33.33 % ↑*
Kirkman 2014[31]	M and F;<br><br>Ex<br>48 ± 18<br>Con<br>58 ± 15	Ex<br>46 ± 54<br><br>Con<br>66 ± 47	Ex (n = 9)<br><br>Con (n = 10)	Ex: progressive resistance training during HD;<br>12 weeks;<br>3 days/week;	6MWT	Ex: 7.33 % ↑<br>Con: 13.04 % ↑

				80 % of their predicted 1RM<br><br>Con: stretching during HD		
Kouidi 1997[48]	M and F;<br><br>Ex<br>49.6 ± 12.1<br>Con<br>52.8 ± 10.2	Ex<br>70.8 ± 58.8<br><br>Con<br>74.4 ± 64.8	Ex (n = 20)<br><br>Con (n = 11)	Ex: stationary cycling, walking or jogging, calisthenics, aerobics, swimming and/or game sports (basketball, football);<br>non-dialysis days;<br>6 months;<br>3 days/week;<br>90 min;<br>50-60 % VO2max<br><br>Con: usual care	VO_2max	Ex: 38.09 % ↑*<br>Con: 1.24 % ↓
Painter 2002[62]	M and F;<br><br>Ex<br>55.9 ± 15.15<br>Con<br>52.8 ± 16.8	Ex<br>33.7 ± 35.6<br><br>Con<br>40.2 ± 62.4		Ex: cycling during HD;<br>5 months;<br>3 days/week;<br>30 min;<br>12 – 14 RPE<br><br>Con: usual care	$peakVO_2$	Ex: 13.3 % ↑*<br>Con: 0.5 % ↑
DePaul 2002[32]	M and F;<br><br>Ex<br>55 ± 16<br>Con<br>54 ± 14	Ex<br>50.4 ± 57.6<br><br>Con<br>55.2 ± 54.0	Ex (n = 20)<br><br>Con (n = 18)	Ex: cycling during HD + strength training for hamstrings and quadriceps muscle groups before or after HD;<br>12 weeks;<br>3 days/week;<br>30 min;<br>13 RPE<br><br>Con: non-progressive, non-resisted, low-intensity range-of-motion exercises of the lower extremities and free upper extremity during HD;<br>12 weeks;<br>3 days/week;<br>30 min	6MWT	Ex: 0.87 % ↑<br>Con: 0.94 % ↑
Konstantinidou 2002[44]	M and F;<br><br>group A<br>46.4 ± 13.9<br>group B<br>48.3 ± 12.1<br>group C<br>51.4 ± 12.5<br>group D<br>50.2 ± 7.9	group A<br>78 ± 62<br>group B<br>72 ± 66<br>group C<br>62 ± 37<br>group D<br>79 ± 86	group A<br>(n = 16)<br>group B<br>(n = 10)<br>group C<br>(n = 10)<br>group D<br>(n = 12)	group A:<br>guided exercise program;<br>non-dialysis days;<br>6 months;<br>3 days/week;<br>60 min;<br>60–70% HRmax<br><br>group B:<br>during HD	$peakVO_2$	group A: 42.77 % ↑*<br>group B: 23.93 % ↑*<br>group C: 17.28 % ↑*<br>group D: 3.07 % ↓

				30 min cycling + 30 min exercises for strength and flexibility<br>6 months;<br>3 days/week;<br>70% HRmax<br><br>group C:<br>home-based program of 30 min cycling + simple flexibility and muscular extension exercises<br>6 months;<br>5 days/week;<br>50–60% HRmax<br><br>group D:<br>usual care		
van Vilsteren 2005[34]	M and F;<br><br>Ex<br>52 ± 15<br>Con<br>58 ± 16	Ex<br>38.6 ± 48.9<br><br>Con<br>46.8 ± 52.9	Ex (n = 53)<br><br>Con (n = 43)	Ex: pre-dialysis strength training programme + cycling (during HD) and exercise counselling<br>12 weeks;<br>2-3 days/week<br>mean duration:<br>strength training:<br>16.78 min<br>cycling:<br>49.86 min<br>12 – 16 RPE<br><br>Con: usual care	STS-10<br><br><br>$peakVO_2$	Ex: 22.36 % ↓*<br>Con: 0.25 % ↓<br><br>Ex: 10.14 % ↑<br>Con: 0.46 % ↑
Kopple 2007[63]	M and F;<br><br>group 1<br>45.9 ± 4.1<br>group 2<br>46.0 ± 2.7<br>group 3<br>42.7 ± 3.8<br>group 4<br>41.3 ± 3.3	group 1<br>45.9 ± 14.1<br>group 2<br>51.9 ± 12.4<br>group 3<br>38.3 ± 5.8 group 4<br>51.4 ± 21.0	group 1<br>(n = 10)<br>group 2<br>(n = 15)<br>group 3<br>(n = 12)<br>group 4<br>(n = 14)	Group 1: cycling during HD;<br>5 months:<br>3 days/week;<br>30 min;<br>50% $peakVO_2$<br><br>Group 2: strength training; before HD;<br>5 months:<br>3 days/week;<br>75% of the 5-RM<br><br>Group 3: endurance + strength training; a combination of approximately one half of the group 1 work effort and also one half of the group 2 work effort; before and during HD;<br>5 months:<br>3 days/week<br><br>Group 4: usual care	CRP	group 1: 44.44 % ↓<br>group 2: 20.00 % ↑<br>group 3: 26.09 % ↑<br>group 4: 33.33 % ↑

Kouidi 2009[38]	M and F;<br><br>Ex<br>54.6 ± 8.9<br>Con<br>53.2 ± 6.1	Ex<br>75.6 ± 44.4<br><br>Con<br>74.4 ± 46.8	Ex (n = 30)<br><br>Con (n = 29)	Ex: cycling + strength training during HD;<br>10 months;<br>3 days/week;<br>90 min;<br>13 RPE<br><br>Con: usual care	peakVO2	Ex: 30.49 % ↑[**]<br>Con: 1.19 % ↓
Koh 2010[52]	M and F;<br><br>group 1<br>52.3 ± 10.9<br>group 2<br>52.1 ± 13.6<br>group 3<br>51.3 ± 14.4	group 1<br>32.1 ± 26.7<br>group 2<br>37.0 ± 31.1<br>group 3<br>25.8 ± 22.2	group 1<br>(n = 15)<br>group 2<br>(n = 15)<br>group 3<br>(n = 16)	group 1: cycling during HD;<br>6 months;<br>3 days/week;<br>30 min;<br>13-14 RPE<br><br>group 2: walking at home;<br>6 months;<br>3 days/week;<br>30 min;<br>13-14 RPE<br><br>group 3: usual care	6MWT	group 1: 13.61 % ↑<br>group 2: 11.04 % ↑<br>group 3: 4.87 % ↑
Afshar 2010[64]	M;<br>group 1<br>50.7 ± 21.06<br>group 2<br>51 ± 16.4<br>group 3<br>53 ± 19.4	group 1<br>25.71 ± 7.61<br>group 2<br>24.86 ± 18.69<br>group 3<br>24.86 ± 15.44	group 1<br>(n = 7)<br>group 2<br>(n = 7)<br>group 3<br>(n = 7)	group 1: cycling during HD;<br>8 weeks;<br>3 days/week;<br>30 min;<br>12-16 RPE<br><br>group 2: resistance training (ankle weights for knee extension, hip abduction, and flexion);<br>8 weeks;<br>3 days/week;<br>30 min;<br>15–17 RPE<br><br>group 3: usual care	CRP	group 1: 83.85 % ↓[**]<br>group 2: 67.89 % ↓[*]<br>group 3: 1.47 % ↑
Ouzouni 2009[39]	M and F;<br><br>Ex<br>47.4 ± 15.7<br>Con<br>50.5 ± 11.7	Ex<br>92.4 ± 84<br><br>Con<br>103.2 ± 72	Ex (n = 19)<br><br>Con (n = 14)	Ex: cycling + strength training during HD;<br>10 months;<br>3 days/week;<br>60-90 min<br>13-14 RPE<br><br>Con: usual care	peakVO$_2$	Ex: 21.05 % ↑[*]<br>Con: 0.99 % ↓
Orcy 2012[65]	M and F;<br><br>group 1<br>56.9 ± 14.8<br>group 2<br>55.8 ± 18.3	group 1<br>22.5<br><br>group 2<br>23	group 1<br>(n = 13)<br><br>group 2<br>(n = 13)	group 1: 20 min of cycling during HD;<br>10 min of resistance training during HD;<br>10 weeks;<br>3 days/week;<br>13-14 RPE	6MWT	group 1: 9.01 %↑[*]<br>group 2: 4.48 %↓

				group 2: 30 min of resistance training during HD;<br>10 weeks;<br>3 days/week;<br>13-14 RPE		
Tao 2015[41]	M and F;<br><br>Ex<br>53.02 ± 11.62<br>Con<br>56.68 ± 9.67	Ex<br>83.46 ± 61.37<br><br>Con<br>84.70 ± 70.55	Ex (n = 57)<br><br>Con (n = 56)	Ex: flexibility and strength exercise before HD;<br>6 weeks;<br>1 day/week;<br>20 min;<br>RPE 12-13<br>+<br>nurse case management on home exercise;<br>1 day/week;<br>20 min<br><br>Con: flexibility and strength exercise before HD;<br>6 weeks;<br>1 day/week;<br>20 min;<br>RPE 12-13	STS-10	Ex: 21.03 % ↓**<br>Con: 16.24 % ↓*
Carmack 1995[66]	M and F;<br><br>All subjects<br>44.09	All subjects<br>29.52	Ex (n = 10)<br><br>Con (n = 11)	Ex: cycling during HD;<br>10 weeks;<br>3 days/week;<br>25 min<br><br>Con: usual care	peakVO_2	Ex: 34.58 % ↑*<br>Con: 9.0 % ↑
Deligiannis 1999[37]	M and F;<br><br>group 1<br>46.4 ± 13.9<br>group 2<br>51.4 ± 12.5<br>group 3<br>50.2 ± 1.9	group 1<br>78±62<br>group 2<br>62±37<br>group 3<br>79±86	group 1<br>(n = 16)<br>group 2<br>(n = 10)<br>group 3<br>(n = 12)	group 1:<br>10-min warm-up on a cycle ergometer or treadmill, a 50-min intermittent aerobic exercise program, including calisthenics, steps and flexibility exercises and a 10-min cooldown period;<br>non-dialysis days;<br>6 months;<br>3 days/week;<br>90 min;<br>60-70% HRmax<br><br>group 2:<br>cycling at home;<br>6 months;<br>5 days/week;<br>30 min;<br>50-60% HRmax +<br>simple flexibility and muscular extension exercises	VO_2max	group 1: 42.8 % ↑**<br>group 2: 17.3 % ↑*<br>group 3: 3.07 % ↓

				group 3:<br>usual care		
Tsuyuki 2003[67]	M and F;<br><br>Ex<br>40.1 ± 11.9<br>Con<br>39.7 ± 10.7	Ex<br>25.2<br><br>Con<br>32.4	Ex<br>(n = 17)<br><br>Con<br>(n = 12)	Ex: combination training of stationary cycling, walking and jogging;<br>non-dialysis days;<br>20 weeks;<br>2-3 days/week;<br>30 min;<br>50-60% of the peak heart rate<br><br>Con: usual care	$peakVO_2$	Ex: 25.58 % ↑[**]<br>Con: 2.25 % ↓
Segura-Ortí 2009[46]	M and F;<br><br>group 1<br>53.5 ± 18.0<br>group 2<br>60.1 ± 16.9	group 1<br>37.3 ± 34.9<br><br>group 2<br>53.7 ± 42.0	group 1<br>(n = 17)<br><br>group 2<br>(n = 18)	group 1:<br>during HD - four progressive isotonic and isometric resistance exercises that targeted major muscle groups of the lower extremities;<br>6 months;<br>3 days/week;<br>25 min;<br>12-14 RPE<br><br>group 2:<br>cycling during HD;<br>6 months;<br>3 days/week;<br>25 min;<br>11 RPE	STS-10<br><br><br><br>6MWT	group 1: 22.31 % ↓[**]<br>group 2: 6.37 % ↓<br><br><br>group 1: 11.21 % ↑[**]<br>group 2: 8.95 % ↑
Johansen 2006[68]	M and F;<br><br>Ex<br>54.4 ± 13.6<br>Con<br>56.8 ± 13.8	Ex<br>33.0<br><br>Con<br>25.5	Ex<br>(n = 20)<br><br>Con<br>(n = 20)	Ex: resistance exercise using ankle weights during HD:<br>60% of 3RM for two sets of 10 repetitions; increased to three sets as tolerated; three sets with correct technique – increase weight;<br>knee extension, hip flexion and abduction, ankle dorsiflexion and plantarflexion;<br>12 weeks;<br>3 days/week<br><br>Con: usual care	STS-10	Ex: 16.11 % ↓<br>Con: 0.66 % ↓
Daniilidis 2004[40]	M and F;<br><br>Ex<br>46.7 ± 13.8<br>Con<br>53.7 ± 7.1	Ex<br>78 ± 62.4<br><br>Con<br>72 ± 81.8	Ex<br>(n = 18)<br><br>Con<br>(n = 14)	Ex: supervised interval aerobic exercise: steps, treadmill, gymnastics, swimming and ball games;<br>non-dialysis days;	$peakVO_2$	Ex: 42.77 % ↑[*]<br>Con: 2.45 % ↓

				6 months;<br>3 days/week;<br>60 min;<br>75%-85% of the estimated peak heart rate<br><br>Con: usual care		
Pellizzaro 2013[45]	M and F;<br><br>Ex<br>48.9±10.1<br>Con<br>51.9±11.6	Ex<br>54<br><br>Con<br>54	Ex<br>(n = 14)<br><br>Con<br>(n = 14)	Ex: 3 sets of 15 repetitions of knee extension;<br>10 weeks;<br>3 days/week;<br>50% of 1RM<br><br>Con: usual care	6MWT	Ex: 6.84 % ↑*<br>Con: 0.07 % ↑
Dobsak 2012[69]	M and F;<br><br>Ex<br>58.2 ± 7.2<br>Con<br>60.1 ± 8.2	Ex<br>49.2 ± 25.2<br><br>Con<br>49.2 ± 27.6	Ex<br>(n = 11)<br><br>Con<br>(n = 10)	Ex: cycling during HD;<br>20 weeks;<br>3 days/week;<br>20-40 min;<br>60% peak workload<br><br>Con: usual care	6MWT	Ex: 15.02 % ↑**<br>Con: 3.32 % ↓
Reboredo 2015[36]	M and F;<br><br>Ex<br>50.7 ± 10.7<br>Con<br>42.2 ± 13.0	Ex<br>39.6 ± 40.8<br><br>Con<br>57.6 ± 52.8	Ex<br>(n = 12)<br><br>Con<br>(n = 12)	Ex: lower-limb stretching exercises (10 min), low-intensity cycling (5 min), moderate-intensity cycling (35 min) and cool down (3 min) during HD;<br>12 weeks;<br>3 days/week;<br>43 min;<br>4-6 RPE<br><br>Con: usual care	peakVO_2	Ex: 12.4 % ↑*<br>Con: 9.2 % ↓
Frih 2017[42]	M;<br><br>Ex<br>64.2 ± 3.4<br>Con<br>65.2 ± 3.1	Ex<br>72.7 ± 12.7<br><br>Con<br>73.6 ± 13.4	Ex<br>(n = 21)<br><br>Con<br>(n = 20)	Ex: resistance training + aerobic training (cycling + walking)<br>16 weeks;<br>4 days/week;<br>40 min;<br>5-6 RPE (Borg 10 grade scale);<br>60% of 1RM<br><br>Con: usual care	6MWT<br><br><br>STS-10	Ex: 15.94 % ↑*<br>Con: 1.56 % ↓<br><br>Ex: 16.2 % ↓*<br>Con: 0.95 % ↑
Valenzuela 2018[43]	M and F;<br><br>Ex<br>68 ± 13<br>Con<br>68 ± 11	Ex<br>84 ± 60<br><br>Con<br>60 ± 48	Ex<br>(n = 27)<br><br>Con<br>(n = 40)	Ex: resistance exercises and cycling during HD;<br>14 weeks;<br>3 days/week;<br>50 min;<br>RPE 13	6MWT<br><br><br><br>STS-10	Ex: 11.05 % ↑**<br>Con: 3.23 % ↓<br><br><br>Ex: 22 % ↓**<br>Con: 6 % ↑

				Con: usual care		
Abreu 2017[33]	M and F;<br><br>All subjects<br>46.4 ± 14.6	ND	Ex<br>(n = 25)<br><br>Con<br>(n = 19)	Ex: resistance training during HD;<br>4 lower limb exercises with ankle cuffs and elastic band resistance;<br>3 sets of 10 rep.;<br>12 weeks;<br>3 days/week;<br>30 min;<br>60% of 1RM<br><br>Con: usual care	CRP	Ex: 24.68 % ↓<br>Con: 1.64 % ↓

Abbreviations: Ex experimental group, Con control group, RM repetition maximum, CRP C-reactive protein, STS-10 ten repetition sit-to-stand test, 6MWT six-minute walk test, HD hemodialysis, M male, F female, RPE rate of perceived exertion, HR heart rate, n number of subjects, SD standard deviation, ND no data. *, $p < 0.05$ indicates significant within-group difference. **, $p < 0.01$ indicates significant within-group difference.
